# Microbiota-Orientated Treatments for Major Depression and Schizophrenia

**DOI:** 10.3390/nu12041024

**Published:** 2020-04-08

**Authors:** Guillaume B. Fond, Jean-Christophe Lagier, Stéphane Honore, Christophe Lancon, Théo Korchia, Pierre-Louis Sunhary De Verville, Pierre-Michel Llorca, Pascal Auquier, Eric Guedj, Laurent Boyer

**Affiliations:** 1 Hôpitaux Universitaires de Marseille, Department de Psychiatrie universitaire, EA 3279: Aix-Marseille Université, CEReSS—Health Service Research and Quality of Life Center, 27 Boulevard Jean Moulin, 13005 Marseille, France; stephane.honore@ap-hm.fr (S.H.); christophe.lancon@ap-hm.fr (C.L.); theo.korchia@ap-hm.fr (T.K.); deverville.pierrelouis@gmail.com (P.-L.S.D.V.); pascal.auquier@univ-amu.fr (P.A.); laurent.boyer@ap-hm.fr (L.B.); 2 Aix Marseille University, Institut de Recherche pour le Développement, Microbes Evolution Phylogeny and Infection, Assistance Publique Hôpitaux de Marseille, Institut Hospitalo Universitaire Méditerranée Infection, 13005 Marseille, France; JeanChristophe.LAGIER@ap-hm.fr; 3 CHU de Clermont-Ferrand, F-63000 Cllermont-Ferrand, France; pmllorca@chu-clermontferrand.fr; 4 Aix-Marseille Université, CNRS, Ecole Centrale de Marseille, UMR 7249, Institut Fresnel, Département de médecine nucléaire, CERIMED, Aix-Marseille Université, F-13005 Marseille, France; eric.guedj@ap-hm.fr

**Keywords:** psychiatry, schizophrenia, depression, microbiota, transplantation

## Abstract

Background and significance. There is a need to develop new hypothesis-driven treatment for both both major depression (MD) and schizophrenia in which the risk of depression is 5 times higher than the general population. Major depression has been also associated with poor illness outcomes including pain, metabolic disturbances, and less adherence. Conventional antidepressants are partly effective, and 44% of the subjects remain unremitted under treatment. Improving MD treatment efficacy is thus needed to improve the SZ prognosis. Microbiota-orientated treatments are currently one of the most promising tracks. Method. This work is a systematic review synthetizing data of arguments to develop microbiota-orientated treatments (including fecal microbiota transplantation (FMT)) in major depression and schizophrenia. Results. The effectiveness of probiotic administration in MD constitutes a strong evidence for developing microbiota-orientated treatments. Probiotics have yielded medium-to-large significant effects on depressive symptoms, but it is still unclear if the effect is maintained following probiotic discontinuation. Several factors may limit MD improvement when using probiotics, including the small number of bacterial strains administered in probiotic complementary agents, as well as the presence of a disturbed gut microbiota that probably limits the probiotics’ impact. FMT is a safe technique enabling to improve microbiota in several gut disorders. The benefit/risk ratio of FMT has been discussed and has been recently improved by capsule administration. Conclusion. Cleaning up the gut microbiota by transplanting a totally new human gut microbiota in one shot, which is referred to as FMT, is likely to strongly improve the efficacy of microbiota-orientated treatments in MD and schizophrenia and maintain the effect over time. This hypothesis should be tested in future clinical trials.

## 1. Introduction

Major depression (MD) is described as “a global crisis” by the World Health Organization (WHO) [1]. Major depression can affect anyone from young people to seniors, and it is one of the most widespread illnesses, often co-existing with other serious illnesses [2]. According to the WHO, MD was ranked as the third leading cause of the global burden of disease in 2004 and will likely have moved to the first place by 2030 [3]. It is now estimated that 350 million people are affected by MD worldwide, which poses a significant health and economic burden to society [4,5,6]. In 2016, MD was the first source of disability, accounting for 1059 worldwide disability-adjusted life years (DALYs)/100,000 habitants, thereby noticeably preceding ischemic and hemorrhagic stroke (787 and 923 respectively), hypertensive heart disease (242), Alzheimer disease (470), cancers (liver (295), colon (249), breast (208), and HIV (169)) [7]. Major depression was responsible for 48.7% of all worldwide DALYs related to mental and substance use disorders [7]. This alarming figure is a wakeup call for researchers and should encourage them to address this global non-communicable disease. 

Major depression is heterogeneous and improving its treatment may require isolating more specific subgroups in the so-called precision medicine approach. Major depressionv has been identified as a frequent comorbidity of other major psychiatric disorders including schizophrenia (SZ). A half of SZ patients have been identified with MD that has been associated with impaired quality of life which suggests a 5 times higher risk of MD in this population compared to non-SZ individuals. Yet MD remains poorly diagnosed and poorly treated in this population [8,9,10]. Some studies suggest that MD-SZ may be different from non-SZ MD with lower placebo response and higher impact on functioning [9,11,12,13]. Major depression in schizophrenia (MD-SZ) has been also associated with other poor illness outcomes including pain, metabolic disturbances, less adherence and lower quality of life [8,14,15]. Treating depression is thus needed to improve the SZ prognosis [16]. Conventional antidepressants are partly effective, but 44% of the subjects remain unremitted under treatment [9]. Yet, funding for research directed to improving diagnosis and treatment of MD-SZ is sadly lacking [17].

Though conventional treatments have improved MD prognosis, they still remain unsatisfactory. The response rate of antidepressants amounts to only 17.7% in the general population [18]. An explanation for this high rate of non-response and relapses relies on the observation that current pharmacological treatments are primarily based on the monoaminergic hypothesis, without involving the personalized medicine approach. According to this hypothesis, MD is principally due to the fact of a deficit of three neurotransmitters in the brain (i.e., serotonin, norepinephrine, and dopamine). All current antidepressants target serotonin, norepinephrine, or dopamine deficits. The high rate of therapeutic failure in psychiatry can most likely be accounted for by the limitations pertaining to brain-orientated treatments. Current treatments do improve neurotransmitters deficits, yet without addressing the source of these deficits. This may explain the high relapsing rates and chronic illness course. 

**Figure 1:**
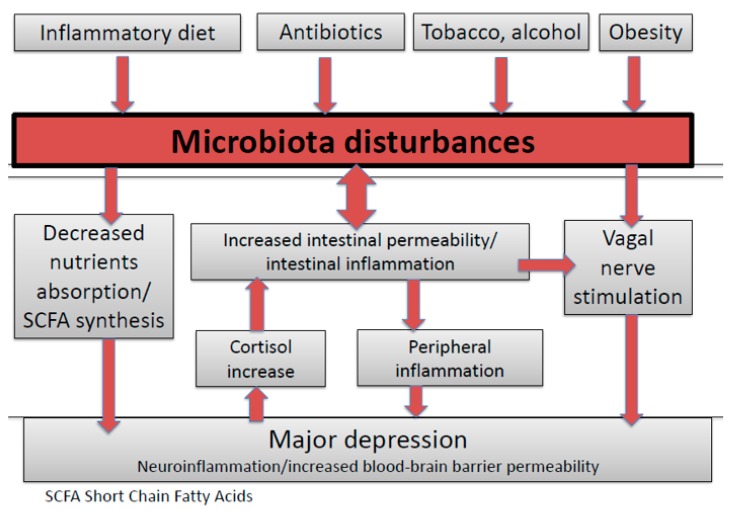
The gut–brain axis in major depression.

The key to breaking the deadlock of SZ-MD treatment may be found in the intestinal microbiota [19]. The links between gut microbiota disturbances and brain dysfunction have clearly been demonstrated in rodents. The so-called “gut-brain axis” has already been extensively described in humans with six pathways [19,20]: vagal nerve stimulation; inflammation and cytokine modulation; decreased gut permeability; short-chain fatty acid and neurotransmitter synthesis; nutrient absorption; Hypothalamic–pituitary–adrenal (HPA) stress axis (cortisol) modulation (Figure 1). Moreover, microbiota dysfunctions have been associated with peripheral immune inflammation as well as neuro-inflammation (also called microglia activation) [21].

Several clues indicate that targeting microbiota may be particularly relevant in schizophrenia (SZ). Schizophrenia patients are treated by antipsychotics that induce gastrointestinal disorders (including constipation) that may impact their gut microbiota. More than one quarter of SZ stabilized outpatients have abdominal obesity, which is a clinical marker of disturbed microbiota, and MD has been found to be the best predictor of rapid high weight gain in SZ [14]. Abnormal bacterial markers have been identified in the blood of SZ patients [22,23]. Emerging data show that about 30% of SZ people have elevated antigliadin antibodies (AGA) of the IgG type, representing a possible subgroup of schizophrenia patients with increased gut permeability [24]. Also, recent data have shown a high correlation of IgG-mediated antibodies between the periphery and cerebral spinal fluid in schizophrenia but not healthy controls, particularly AGA IgG suggesting that these antibodies may be crossing the blood-brain barrier with resulting neuroinflammation [25]. Schizophrenia has been extensively associated with other abnormal translational markers, suggesting an increased gut permeability in this illness [23,25,26,27,28,29]. More than one in five SZ patients are identified with metabolic syndrome [30], and one-third with chronic low-grade peripheral inflammation [31,32,33,34]. This inflammation is a good marker of central inflammation and has also been associated with SZ-MD [35].

Our hypothesis is that replacing the whole microbiota of SZ-MD patients (the so-called fecal microbiota transplantation (FMT)) may improve their mental and physical health, and more specifically their depressive symptoms and quality of life. Schizophrenia combined with MD and/or inflammation may be a target of choice for microbiota-orientated therapies.

The objective was to synthetize current data for testing microbiota-orientated treatments and to explore the benefit/risk ratio of FMT in major depression and schizophrenia.

## 2. Methods

This meta-analysis was based on the Preferred Reporting Items for Systematic reviews and Meta-Analysis (PRISMA) criteria [36] (Figure 1). Medline^®^ database was explored from its inception to March, 22th 2020 without language restriction. The research paradigm was: (depression OR schizophrenia) AND (gut microbiota). The references of each article were also checked. Medline is considered as the database of highest quality level. The associated articles were also explored. Scopus^®^ and ScienceDirect^®^ databases were explored with the same strategy (limited to research articles and research reviews and human studies). Two reviewers (GF and LB) decided on eligibility and extracted data from included studies. As this review involved data from published studies, an institutional review board approval was not required.

### 2.1. Criteria for Included Studies:

- Design: Human observational and interventional studies and meta-analyses including human data;
- Exploring the association between microbiota disturbances (or irritable bowel syndrome) and major depression or schizophrenia defined by a DSM or ICD-based diagnostic tool (structured clinical interview) OR assessing the efficacy of a microbiota-orientated therapy (probiotics or fecal microbiota transplantation).

### 2.2. Exclusion Criteria:

- animal studies;
- studies including no individuals with major depression or schizophrenia;
- case reports;
- reviews.

## 3. Results

Fourteen studies were included in the present review.

### 3.1. Microbiota-Orientated Therapies and Their Interest for Major Depression 

Irritable bowel syndrome is considered as a paradigmatic microbiota-induced illness. We have published a meta-analysis suggesting that patients with irritable bowel syndrome were at higher risk of major depression [37], confirming the potential causal or bilateral relationship between microbiota disturbances and major depression. Several studies have shown microbiota disturbances in patients with major depression; these disturbances are summarized in Table 1 [38,39,40,41,42,43,44,45,46,47,48,49,50]. 

The effectiveness of probiotic administration in MD constitutes a strong evidence for developing microbiota-orientated treatments in this indication. Probiotics have yielded medium-to-large significant effects in the setting of depression (d = −0.73 (95% CI = −1.02–−0.44)) in a recent meta-analysis [51]. Approximately half of all existing studies were published over the past two years, reflecting the rapidly growing interest in this area. At the time of this submission, 29 studies involving 3088 participants were published so far. Duration of probiotic administration across trials ranged from 8 days to 45 weeks, whereas it is still unclear if the effect is maintained following probiotic discontinuation.

Two factors may limit MD improvement when probiotics are administered: (1) the small number of bacterial strains administered in probiotic complementary agents (often only one to five bacterial strains including *Lactobacilli*, either alone or in combination with *Bifidobacterium*), and (2) the presence of a disturbed gut microbiota that limits probiotics’ efficacy (the so-called gut microbiota “resilience”). Cleaning up the gut microbiota and transplanting a totally new human gut microbiota in one shot (the so-called fecal microbiota transplantation) would thus strongly improve the effect size.

### 3.2. Fecal Microbiota Transplantation’s Effectiveness in Non-Psychiatric Diseases

If MD is actually associated with microbiota dysfunctions, replacing disturbed microbiota by a healthy one appears to be one of the most promising approach to improve MD [52]. FMT has been described as “the ultimate probiotic” as it provides an entire microbiome to the recipient. This therapy delivers a much greater number and diversity of bacteria than any current commercially available preparation. In the past decade, there has been a heightened interest in the use of this therapy [53], predominantly driven by increasing rates of recurrent *Clostridium difficile* infection [54,55,56]. 

This procedure was proven associated with 87%–100% clinical resolution of recurrent or refractory *C. difficile* infections [56,57,58,59,60]. This impressive success rate is presumably due to the ability of the transplanted bacteria to recolonize/occupy the missing components/niches of the normal intestinal microbiota thus removing the microbial niche that *C. difficile* would otherwise exploit.

In addition to this main application, FMT has demonstrated promising results in other diseases as well such as ulcerative colitis [61,62] or inflammatory bowel diseases [63].

### 3.3. Fecal Microbiota Transplantation’s Safety in Non-Psychiatric Diseases

No serious adverse event related to FMT has been reported in the literature. In a recent review, the commonest FMT-attributable adverse event was abdominal discomfort, which was reported in 19 publications [64]. 

There is a potential to transmit infection via contaminated donor stool. The donor stool must therefore undergo microscopy and culture for potential bacterial pathogens, microscopy for ova, cysts and parasites as well as viral studies and C. *difficile* toxin analysis. Blood testing to exclude HIV, Hepatitis B and C and syphilis must be undertaken.

Changes in fecal microbiota have been found in patients with a number gastrointestinal and extra-intestinal diseases. Changes in the microbiome of patients with inflammatory bowel diseases and irritable bowel syndrome are well documented in the literature [65].

There have also been associations between various bowel flora, obesity, and the metabolic syndrome. The association has not been documented as causal, and it appears probably related to the diet consumed by these subjects. It would, however, be prudent to exclude donors with the metabolic syndrome.

SZ patients are already treated with antipsychotics, antidepressants, and other psychotropic drugs that have many side-effects (including sedation, weight gain, neurological disorders, diarrhea, and constipation), the FMT appears as a safe treatment in comparison of the standard treatment for SZ and MD. The risk–benefit balance seems favorable.

### 3.4. Oral Capsules Administration: An Improvement for Fecal Microbiota Transplantation Safety

The oral capsule administration form has proven an equal effectiveness [66] and will prevent the adverse event due to the conventional colonoscopy-delivered upper and lower gastrointestinal routes of FMT, especially bowel perforation over-sedation, aspiration, bleeding, and splenic laceration [67,68]. Some studies reported patient deaths due to the underlying disease, where the patient has not responded to the FMT. Our clinical experience and our 5 years collaboration with patients’ associations has also shown to us that an important rate of the patients and their relatives are waiting for innovating treatments targeting new pathways, with a better tolerance than antipsychotics. In France, the microbiota hypothesis is very popular and highly broadcasted in the media. 

## 4. Conclusions

Cleaning up the gut microbiota by transplanting a totally new human gut microbiota in one shot, which is referred to as FMT, is likely to strongly improve the efficacy and maintains the effect over time. The safety and acceptability have been recently improved with capsule administration that should be evaluated in future clinical trials for the treatment of major depression and schizophrenia. Future trials should confirm the effectiveness and identify responder profiles in the context of personalized medicine. 

## Figures and Tables

**Table 1:** Human studies exploring microbiota disturbances in major depression and the interest of microbiota-orientated therapies.

Author/Date	Sample Size and Study Population (*N*)	Techniques	Major Findings	Interpretation
**Fond et al. 2014 [37]**	10 studies (885 patients and 1384 HCs)	Meta-analysis	Patients with IBS had significant higher anxiety and depression levels than controls (respectively, SMD = 0.76, 95% CI 0.47; 0.69, *p* < 0.01, I2 = 81.7% and SMD = 0.80, 95% CI 0.42; 1.19, *p* < 0.01, I2 = 90.7%). This significant difference was confirmed for patients with IBS-C and -D subtypes for anxiety, and only in IBS-D patients for depression.	Patients with IBS had significantly higher levels of anxiety and depression than HCs.
**Liu et al. 2019 [51]**	29 studies involving 3088 participants	Meta-analysis	Prebiotics did not differ from placebo for depression (d = −0.08, *p* = 0.51) or anxiety (d = 0.12, *p* = 0.11). Probiotics yielded small but significant effects for depression (d = −0.24, *p* < 0.01) and anxiety (d = −0.10, *p* = 0.03). Sample type was a moderator for probiotics and depression, with a larger effect observed for clinical/medical samples (d = −0.45, *p* < 0.001) than community ones. This effect increased to medium-to-large in a preliminary analysis restricted to psychiatric samples (d = −0.73, *p* < 0.001).	There is general support for antidepressant and anxiolytic effects of probiotics, but the pooled effects were reduced by the paucity of trials with clinical samples.
**Ng et al. 2019 [49]**	3 studies	Meta-analysis	No significant difference in schizophrenia symptoms between the group that received probiotic supplementation and the placebo group post-intervention as the standardized mean difference was -0.0884 (95% CI -0.380 to 0.204, *p* = 0.551). Separate analyses were performed to investigate the effect of probiotic supplementation on positive or negative symptoms of schizophrenia alone. In both instances, no significant difference was observed as well.	Based on current evidence, limited inferences can be made regarding the efficacy of probiotics in schizophrenia
**Kiecolt-Glaser et al. 2018 [50]**	43 (N = 86) healthy married couples, ages 24–61 (mean = 38.22)	Translocation of bacterial endotoxin (lipopolysaccharide, LPS) from the gut microbiota	Participants with more hostile marital interactions had higher LPS-binding protein (LBP) than those who were less hostile. Additionally, the combination of more hostile marital interactions with a mood disorder history was associated with higher LBP/sCD14 ratios.	The combination of more hostile marital interactions with a mood disorder history was associated with higher LBP/sCD14 ratios.
**Chen et al. 2018 [44,45]**	10 patients (age: 18–56 years, five women) who had MDD and 10 HCs (age: 24–65 years, five women) matched for sex, age, and BMI	Comparative metaproteomics analysis on the basis of an isobaric tag for relative and absolute quantification coupled with tandem mass spectrometry	279 significantly differentiated bacterial proteins (*p* < 0.05) were detected and used for further bioinformatic analysis. According to phylogenetic analysis, statistically significant differences were observed for four phyla: *Bacteroidetes, Proteobacteria, Firmicutes, Actinobacteria* (*p* < 0.05, for each). Abundances of 16 bacterial families were significantly different between the MDD and healthy controls (*p* < 0.05). Cluster of Orthologous Groups analysis and Kyoto Encyclopedia of Genes and Genomes pathway analysis showed that disordered metabolic pathways of bacterial proteins were mainly involved in glucose metabolism and amino acid metabolism.	Fecal microbiota signatures were altered significantly in MDD patients.
**Peter et al. 2018 [38]**	48 patients with IBS (Rome III criteria, M (SD) age = 42 (15) years, 35 female, 25 diarrhea-dominant, 5 constipation-dominant, and 18 alternating-type IBS)	alpha and beta diversity, correlational analyses of bacterial abundance and comparisons among subgroups defined by thresholds of psychological and IBS symptom variables, and machine learning to identify bacterial patterns corresponding with psychological distress.	Thirty-one patients (65%) showed elevated psychological distress, 22 (31%) anxiety, and 10 depression (21%). Microbial beta diversity was significantly associated with distress and depression (q = 0.036 each, q values are p values false discovery rate-corrected for multiple testing). Depression was negatively associated with *Lachnospiraceae* abundance (Spearman’s ρ = −0.58, q = 0.018). Patients exceeding thresholds of distress, anxiety, depression, and stress perception showed significantly higher abundances of *Proteobacteria* (q = 0.020–0.036). Patients with anxiety were characterized by elevated *Bacteroidaceae* (q = 0.036). A signature of 148 unclassified species accounting for 3.9% of total bacterial abundance co-varied systematically with the presence of psychological distress.	Psychological variables significantly segregated gut microbial features, underscoring the role of brain-gut-microbiota interaction in IBS. A microbial signature corresponding with psychological distress was identified.
**Kelly et al. 2016 [39]**	34 MDD patients and 33 matched HCs	16s rRNA sequencing	Chao1 richness (U = 424, *p* = 0.005), total observed species (U = 441, *p* = 0.002) and phylogenetic diversity (U = 447.5, *p* = 0.001) were decreased in the depressed group. was no difference in Shannon diversity (U = 350, *p* = 0.197). Significant differences in beta diversity between the healthy and depressed groups (Bray-Curtis (*p* = 0.014), unweighted unifrac (*p* = 0.002) and weighted unifrac (*p* = 0.018) were unable to separate groups according to PCoA analysis). The difference of the global microbiota composition from the 16S rRNA data of the depressed and control groups was assessed by ordination. Statistics based on random permutations of the redundancy analysis (RDA) showed that the depressed group is significantly separated at genus level (*p* = 0.03) from the control group. No difference on intestinal permeability, short chain fatty acids, fecal metabolites has been reported.	Depression is associated with decreased gut microbiota richness and diversity
**Lin et al. 2017 [48]**	*N* = 10 MDD	V3–V4 region of the 16S rRNA gene was extracted from the fecal microbial communities in MDD patients, PCR amplified and sequenced on the Illumina Miseq platform	More phylum Firmicutes, less Bacteroidetes, and more genus Prevotella, Klebsiella, Streptococcus and Clostridium XI were found in MDD patients. The changes of the proportion of Prevotella and Klebsiella were consistent with Hamilton depression rating scale.	Prevotella and Klebsiella proportion in fecal microbial communities should be concerned in the diagnosis and therapeutic monitoring of MDD in future.
**Liu et al. 2016 [42]**	*N* = 100 40 with diarrhea-predominant IBS (IBS-D), 15 with depression, 25 with comorbidities of depression and IBS patients, and 20 healthy individuals (controls)	Colonic mucosal inflammation was assayed by immunohistochemical analyses of sigmoid biopsied tissues	Fecal microbiota signatures were similar between patients with IBS-D and depression presented, in that they were less diverse than samples from controls and had similar abundances of alterations. were characterized by high proportions of *Bacteroides* (Type I), *Prevotella* (Type II), or non-dominant microbiota (Type III). Most patients with IBS-D or depression had Type I or Type II profiles (IBS-D had 85% Type I and Type II, depression had 80% Type I and Type II profiles).	Patients with IBS-D and depression have similar alterations in fecal microbiota; these might be related to the pathogenesis of these disorders. 3 microbial profiles in patients could indicate different subtypes of IBS and depression or be used as diagnostic biomarkers
**Jiang et al. 2015 [43]**	46 patients with depression (29 active-MDD and 17 responded-MDD) and 30 healthy controls (HCs).	high-throughput pyrosequencing	Increased fecal bacterial α-diversity was found in the active-MDD (a-MDD) vs. the HC group but not in the responded-MDD (R-MDD) vs. the HC group. *Bacteroidetes, Proteobacteria*, and *Actinobacteria* strongly increased in level, whereas that of *Firmicutes* was significantly reduced in the A-MDD and R-MDD groups compared with the HC group. Despite profound interindividual variability, levels of several predominant genera were significantly different between the MDD and HC groups. Most notably, the MDD groups had increased levels of *Enterobacteriaceae* and *Alistipes* but reduced levels of *Faecalibacterium*. A negative correlation was observed between *Faecalibacterium* and the severity of depressive symptoms.	These findings enable a better understanding of changes in the fecal microbiota composition in such patients, showing either a predominance of some potentially harmful bacterial groups or a reduction in beneficial bacterial genera.
**Kleiman et al. 2015 [41]**	Inpatients with anorexia nervosa at admission (T1; *n* = 16) and discharge (T2; *n* = 10). Patients with anorexia nervosa were compared with healthy individuals who participated in a previous study (HCs).	Genomic DNA was isolated from stool samples, and bacterial composition was characterized by 454 pyrosequencing of the 16S rRNA gene.	Significant changes emerged between T1 and T2 in taxa abundance and beta (between-sample) diversity of patients with anorexia nervosa. Patients with anorexia nervosa had significantly lower alpha (within-sample) diversity than did HCs at both T1 (*p* = 0.0001) and T2 (*p* = 0.016), and differences in taxa abundance were found between anorexia nervosa patients and HCs.	There was evidence of an intestinal dysbiosis in anorexia nervosa and an association between mood and the enteric microbiota in this patient population
**Madan et al. 2020 [47]**	Adult MDD inpatients (N = 111)	16S rRNA gene sequencing and whole genome shotgun sequencing	Depression and anxiety severity shortly after admission were negatively associated with bacterial richness and alpha diversity. Additional analyses revealed a number of bacterial taxa associated with depression and anxiety severity. Gut microbiota richness and alpha diversity early in the course of hospitalization was a significant predictor of depression remission at discharge.	There is a gut microbiota relationship with symptom severity among MDD inpatients as well as a relationship to remission of depression post-treatment.
**Mason et al. 2020 [46]**	N = 70 (60 psychiatric subjects; MDD (comorbid with anxiety), *n* = 38, anxiety only, *n* = 8, MDD only without anxiety, *n* = 14, HCs *n* = 10	Quantitative PCR and 16S rRNA sequencing	Altered microbiota correlated with pre-defined clinical presentation, with *Bacteroides* (*p* = 0.011) and the *Clostridium leptum* subgroup (*p* = 0.023) significantly different between clinical categories. Cluster analysis of the total sample using weighted UniFrac β-diversity of the gut microbiota identified two different clusters defined by differences in bacterial distribution. Cluster 2 had higher *Bacteroides* (*p* = 0.006), and much reduced presence of *Clostridales* (*p* < 0.001) compared to Cluster 1. *Bifidobacterium* (*p* = 0.0173) was also reduced in Cluster 2 compared to Cluster 1. When evaluated for clinical charateristics, anhedonia scores in Cluster 2 were higher than in Cluster 1.	Reduced or absent *Clostridia* was consistently seen in those with depression, independent of the presence of anxiety. Conversely, reduced *Bacteroides* may be more associated with the presence of anxiety, independent of the presence of depression.
**Naseribafrouei** **et al., 2014** [40]	N = 55 (37 MDD, and 18 HCs)	Illumina deep sequencing of 16S rRNA gene amplicons	The order *Bacteroidales* showed an overrepresentation (*p* = 0.05), while the family *Lachnospiraceae* showed an underrepresentation (*p* = 0.02) of Operational Taxonomic Units associated with depression.	Several correlations were found between depression and fecal microbiota.

MDD: Major depressive disorder. IBS: irritable bowel syndrome. HCs: Healthy controls. BMI: body mass index.
